# Efficacy of Xanthan‐Based Chlorhexidine Gel in Peri‐Implant Mucositis Treatment: A Split‐Mouth Randomized Clinical Trial

**DOI:** 10.1111/cid.70137

**Published:** 2026-03-21

**Authors:** Jessica Curcio, Jacopo Lanzetti, Armando Crupi, Giulia Ambrogio, Umberto Gibello, Andrea Roccuzzo, Francesco Pera

**Affiliations:** ^1^ Department of Surgical Sciences – CIR Dental School University of Turin Turin Italy; ^2^ Shanghai Perio‐Implant Innovation Center, Institute of Integrated Oral, Craniofacial and Sensory Research, Shanghai Ninth People’s Hospital, Shanghai Jiao Tong University School of Medicine, College of Stomatology, Shanghai Jiao Tong University, National Center for Stomatology, National Clinical Research Center for Oral Diseases, Shanghai Key Laboratory of Stomatology, Shanghai Research Institute of Stomatology Shanghai China; ^3^ Department of Periodontology School of Dental Medicine, University of Bern Bern Switzerland

**Keywords:** adjunctive treatment, antimicrobials, chlorhexidine, clinical trial, non‐surgical treatment, peri‐implant mucositis

## Abstract

**Objectives:**

To investigate the potential benefits of Xanthan‐based chlorhexidine gel application in addition to professional mechanical plaque removal (PMPR) in the treatment of peri‐implant mucositis (PM).

**Material and Methods:**

Subjects diagnosed with PM were consecutively included in this randomized split‐mouth study. All participants received a single session of PMPR using titanium curettes, followed by the application of an air‐polishing glycine powder device. Implants allocated to the Test group were additionally treated with local delivery of Xanthan‐based chlorhexidine gel. Clinical evaluation was performed at T0 (i.e., baseline), at 30 (T1), 90 (T2) and 180 days (T3) after treatment, while treatment success was evaluated at T2 and T3. Change in bleeding on probing (BoP) was considered as primary outcome measure. A logistic multivariate regression model was developed to explore the predictive role of implant and patient‐level variables on primary outcome measure.

**Results:**

Fifty‐nine patients (mean age: 65.4 ± 8.7 years; 54.2% male; 88.1% non‐smokers) and 182 implants completed the study. At T1, only the Test group displayed a significant reduction in BoP (*p* < 0.001), PPD (*p* = 0.021) and PI (*p* = 0.021) compared to T0, while at T2 and T3 clinical improvements were recorded within both groups without any statistically significant difference between groups (*p* > 0.05). T2 Treatment success as well as the frequency distribution of complete (BoP = 0) and partial (BoP ≤ 1, ≤ 2, ≤ 3) disease resolution did not significantly differ between groups (*p* > 0.05). Multiple regression model revealed that smoking (*p* = 0.008), and implant position (i.e., premolar *p* = 0.009) did significantly affect the primary outcome measure.

**Conclusion:**

The adjunctive use of XanCHX gel did not result in any statistically significant clinical benefit compared to PMPR alone in the treatment of PM up to 6 months, despite the reported clinical positive effects within the first month after treatment.

## 1 Introduction

During the 2017 World Workshop, peri‐implant diseases were defined as biofilm‐associated pathological conditions affecting osteointegrated implants, further classified into peri‐implant mucositis and peri‐implantitis [1, 2, 3]. Peri‐implant mucositis (PM) is an inflammatory and reversible lesion that surrounds the peri‐implant mucosa without loss of supporting bone, clinically characterized by bleeding on gentle probing [2], erythema, swelling, and/or suppuration [4]. It has been estimated that it affects approximately 21%–88% of individuals and 9%–51% of implant sites, with prevalences of 47% and 29%, respectively [5]. PM arises from healthy peri‐implant mucosa due to the buildup of bacterial biofilms around implants [2]. If not properly diagnosed and treated, it may lead to peri‐implantitis, a not reversible inflammatory lesion characterized by a non‐linear accelerating pattern that affects the supporting bone around the implant, which may ultimately lead to implant loss [4, 6].

Experimental studies show that PM can be reversed if proper biofilm management is maintained [7, 8]. Prevention and regression of inflammation around implants can be achieved through proper oral hygiene and an effective supportive care protocol, which may include regular clinical check‐ups, radiographic assessments, oral hygiene instructions and professional mechanical plaque removal (PMPR) [9]. More specifically, at the time being, PMPR combined with oral hygiene instructions is considered the gold standard in managing PM [1, 2, 10]. Nevertheless, numerous experimental studies have demonstrated that peri‐implant tissues exhibit a more severe and prolonged inflammatory response to biofilm compared to periodontal tissues, establishing that attaining peri‐implant health presents greater challenges [11]. Therefore, adjunctive measures such as self‐administration of oral rinse antiseptics (i.e., chlorhexidine) may be considered [1]. Several systematic reviews [12, 13, 14] have analyzed the effect of different adjunctive methods to conventional PMPR for the treatment of PM. Even though the adjunctive use of local antiseptics led to greater reductions in probing depth (PPD) [14] and bleeding on probing (BoP) [12], all these studies consistently concluded that complete disease resolution was not achieved. However, the addition of chlorhexidine to PMPR with different concentrations (0.06% or 0.12% or 0.2%) or in combination with 0.05% cetylpyridinium chloride, as well as in the form of a gel with a concentration of 0.2% has shown a clinical improvement in the treatment of PM, showing a significant reduction in BoP [15]. Moreover, the use of a chlorhexidine‐based gel in combination with xanthan (XanCHX) in addition to PMPR has demonstrated positive clinical results in the treatment of periodontitis [16, 17, 18].

Xanthan‐based gel (Chlosite, Ghimas, Bologna, Italy) contains 0.5% chlorhexidine digluconate, 1% chlorhexidine dihydrochloride, and 0.5% xanthan gel. It is a polymer that creates a three‐dimensional, pseudo‐plastic network with water, forming a stable gel that allows a slow and prolonged release of chlorhexidine, allowing effectiveness for about 30 days. Chlorhexidine digluconate is released immediately upon application, whereas chlorhexidine dihydrochloride is gradually delivered the following days, providing bacteriostatic and bactericidal effects for up to 2 weeks, helping the prevention of recolonization of the treated site.

A systematic review [19] has shown that PMPR combined with the topical use of XanCHX gel leads to a significant reduction in PPD over the 3 months after therapy in patients with periodontitis, suggesting that xanthan improves the effectiveness of chlorhexidine by increasing its thickness and ability to stay longer in the treated site, extending its efficacy up to 4 weeks after application. Consequently, it seems plausible that the additional use of XanCHX gel could improve the effectiveness of PMPR in the treatment of PM.

Therefore, the aim of the present study was to investigate the potential benefits of XanCHX gel in adjunct to PMPR in patients affected by PM.

## 2 Materials and Methods

The study protocol was designed in accordance with the revised Helsinki Declaration (2024) and approved by the Ethical Committee of “A.O.U. Città della Salute e della Scienza” (ref. 0000620). The trial was registered on Clinicaltrial.gov (NCT07047261). All patients were informed about the procedures and provided signed informed consent prior to study enrollment.

### 2.1 Study Design and Group Allocation

This study was conducted as a split‐mouth randomized (1:1 ratio) clinical trial with two parallel groups and a 6‐month follow‐up. Data were reported according to the Consolidated Standards of Reporting (CONSORT) guidelines [20]. Implants were randomly allocated to the test and control groups after PMPR with sealed envelopes by an external investigator (G.A.) not involved either in the intervention nor in the outcomes evaluation.

### 2.2 Hypothesis

The null‐hypothesis (H0) was that no statistically significant difference with respect to the mean change in BoP (%) following PMPR with adjunctive application of XanCHX gel would be detected compared with PMPR alone.

### 2.3 Study Population

Subjects attending the Oral Rehabilitation, Maxillo‐Facial Prosthesis and Implantology Unit of C.I.R. Dental School, A.O.U. Città della Salute e della Scienza of Turin (Italy) between January and December 2024, were consecutively screened for recruitment. One experienced operator (F.P.) evaluated all subjects and was responsible for patients' enrollment process following the assessment of the inclusion and exclusion criteria.

### 2.4 Inclusion Criteria

- Subjects aged > 18 years
- Systemically healthy subjects or those with controlled medical conditions
- Participants with one osseointegrated dental implant per quadrant (i.e., maxillary and/or mandibular) supporting one single crown or with two implants supporting a three‐unit fixed dental prosthesis. In this scenario, in presence of only one implant diagnosed with PM, the affected implant was included within the study while in cases of both implants with PM, the included implant was randomly selected by toss coin
- Implant in function for at least 1 year
- Implants diagnosed with PM, defined as the presence of BoP (more than one spot at a location around implant or a line of bleeding or profuse bleeding) around each implant with no bone loss (< 3 mm) evident on radiographs [1, 21, 22].

### 2.5 Exclusion Criteria

- Systemic disease that might influence treatment outcome (i.e., uncontrolled diabetes mellitus, head and neck radiotherapy, chemotherapy)
- Pregnant or breastfeeding mothers
- Cigarettes smoking > 10 cig./day
- Diagnosis of peri‐implantitis according to EFP [1]
- Untreated periodontal conditions: FMPS > 20% and FMBS > 20%
- Implant mobility
- Implants supporting full‐arch reconstructions
- Allergy to chlorhexidine and/or other gel components

### 2.6 Intervention

**FIGURE 1:**
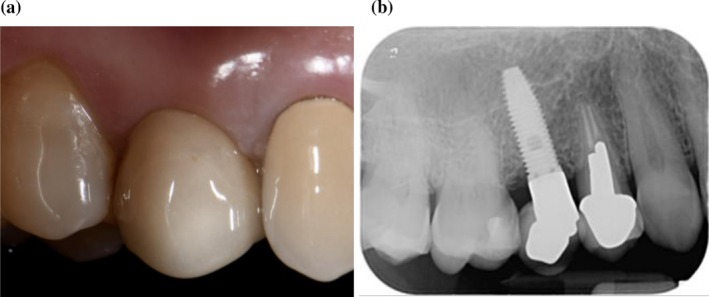
Clinical (a) and radiographic (b) appearance of test implant in regio 1.5 at T0.

**FIGURE 2:**
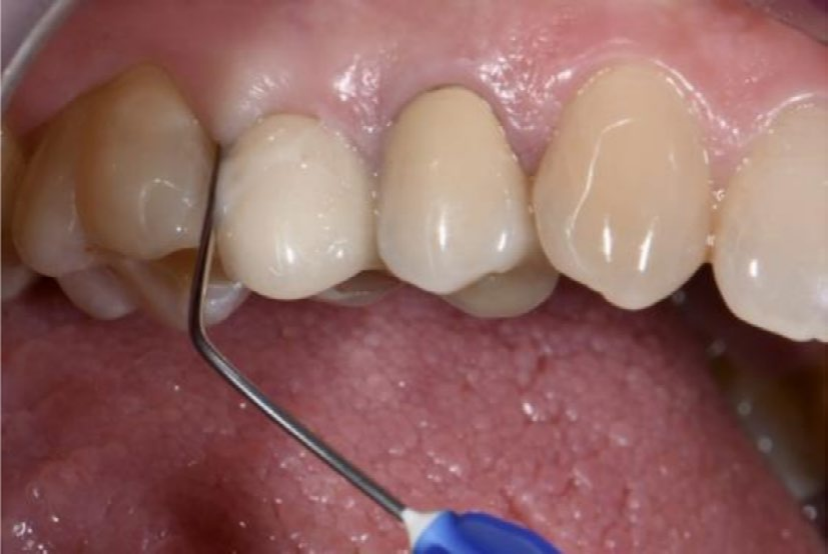
Clinical application of Xanthan and Chlorhexidine gel following PMPR.

**FIGURE 3:**
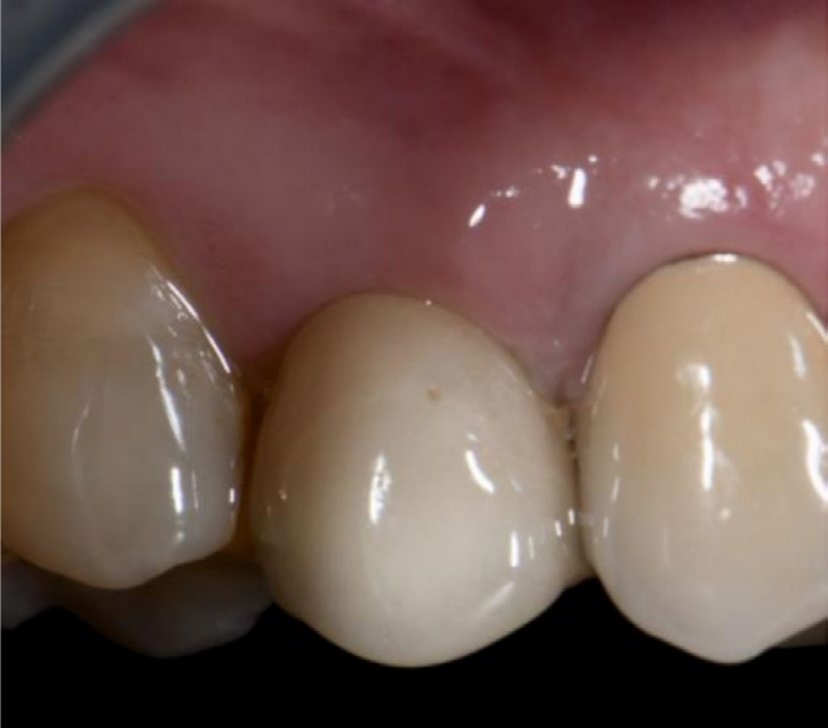
Clinical appearance of test implant in regio 1.5 at T3.

At baseline (T0), after recording clinical parameters, patients received instruction on the use of power‐driven toothbrush and interdental brushes. Thereafter, all selected implants received PMPR using titanium curettes, followed by the application of an air‐polishing device with 25‐μm glycine powder (Airflow Perio; EMS; Nyon; Switzerland). Subsequently, according to the performed randomization, the therapist cleaned and dried the site using compressed air and applied the XanCHX gel on the implant test sites (Figures 1, 2, 3), positioning the blunt needle at the bottom of the pocket and gently extruding the product while moving it out of the site. The control group received PMPR alone. Finally, patients were instructed to avoid from drinking or rinsing for the following hour. All procedures were performed by one experienced RDH (J.C.).

### 2.7 Outcome

Clinical parameters were assessed at baseline (T0), at 30 (T1), 90 (T2), and 180 (T3) days. One experienced and calibrated examiner (J.L.) (Kappa score 0.907;95% confidence interval [CI] 0.894 to 0.999; *p* < 0.01) performed all measurements and recorded data using a North Carolina periodontal probe (UNC 15; Hu‐Friedy, Chicago, IL, USA) at six sites of each selected implant for BoP, PI, PPD and CAL. PISTD and KMW were assessed exclusively at the mid‐buccal aspect of each implant:

Parameters were synthesized as follows:

- Bleeding on Probing (BoP) change was assumed as the primary outcome, considering as clinically relevant a difference of 20% in terms of frequency in BoP+ sites between the two treatment groups at 3‐month follow‐up. BoP was recorded as the percentage of bleeding after gentle probing at sites around the implant [23] (primary outcome).
- Probing depth (PPD), measured from the peri‐implant mucosal margin to the base of the sulcus, applying a force of 0.2 N (20 g) [24]
- Presence or absence of plaque (PI) [25]
- Keratinized mucosal width (KMW), measured from the mucogingival junction to the gingival margin surrounding the implant [26, 27]
- Peri‐implant soft‐tissue dehiscence (PISTD), measured as the exposure of the prosthetic abutment or the implant neck or the implant surface [26, 28, 29]
- Clinical attachment level (CAL), measured from the crown‐abutment junction to the bottom of the peri‐implant pocket [30, 31]

### 2.8 Treatment Success

Treatment success was defined as the presence of a maximum of one site BoP+ per implant [32] at T2 and T3. All patients whose implants did not meet the success criteria at T3 were informed and additional treatment was offered according to their needs.

### 2.9 Data Analysis

The sample size was calculated using the JPower tool of Jamovi software (version 2.3.28.0) considering BoP change at T2 as the primary outcome. More specifically, a clinically relevant difference of 20% in terms of frequency in BoP positive sites between the two treatment groups at 3‐month follow‐up was considered statistically significant [33]. The sample size calculation suggested including 55 patients (91 implants per group; 182 in total) with a power of 80% and an *α* = 0.05. Considering a potential drop‐out rate of 5%, 59 patients were consequently randomized.

Both patient and implant were statistical units due to the two‐level structure of data. Continuous parameters were described using mean, standard deviation and 95% confidence intervals. Categorical variable (i.e., treatment success) was described using proportions. Changes of parameters between timepoints (T0–T1, T0–T2, T0–T3) were calculated and described in the same way.

Multi‐level simple linear regression using generalized estimation equations (GEE) were conducted to assess the effect of treatment group, time and interaction on different clinical parameters (BoP, PD, PI, KMW, CAL) taking into account the within‐patient correlation of multiple implants. A binary logistic regression model was applied for the analysis of disease resolution. Exchangeable correlation matrix with robust Huber‐White estimators were used because better goodness of fit indexes (Akaike) than unstructured one. Multiple pairwise intra and intergroup comparisons were performed correcting by Bonferroni's criteria. Overall changes of clinical parameters (T0–T3) were considered as dependent variables and the same previous methodology was used for assessing the influence of independent patient‐level variables, positional and baseline implant‐level measurements. Simple and multiple linear models provided non‐adjusted and adjusted beta coefficients and 95% confidence intervals from Wald's Chi2 statistic. Disease resolution at T3 was considered the dependent variable of similar approach using simple and multiple logistic models providing raw and adjusted OR. Partial resolution rates (i.e., number of implants with BoP ≤ 1, ≤ 2, ≤ 3) were compared between groups with similar models. Significance level used in all analysis was 5% (alpha = 0.05). The statistical analysis was performed by a professional biostatistician blinded to the provided treatment using a dedicated software (STATA BE, version 17.1, StataCorp LP, College Station, TX).

## 3 Results

### 3.1 Subjects Accountability, Population and Implants Characteristics

**FIGURE 4:**
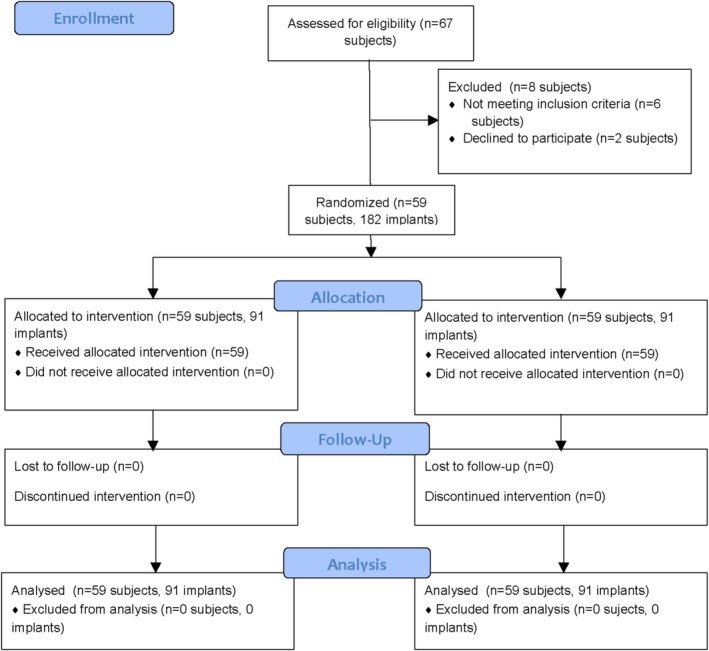
Study flow‐chart.

Out of the 67 patients originally enrolled, 59 (mean age: 65.4 ± 8.7 years; 54.2% male; 88.1% non‐smokers) were randomized and completed the study. The study flow chart is reported in Figure 4. History of periodontitis was reported in 38 (64.4%) subjects. Mean FMPS and FMBS were 11.4 ± 5.2 and 11 ± 5.5, respectively. Each patient had on average 3.08 ± 1.30 implants (range 2–8 implants/patients; total: 182 implants). With respect to implant position (i.e., anterior vs. premolar vs. molar) and arch (i.e., lower vs. upper), no statistically significant differences was detected between groups (*p* > 0.05). All peri‐implant clinical parameters, except for KMW and CAL, were comparable between test and control group (*p* > 0.05). Details of baseline patients and implants characteristics are provided in Table 1.

**TABLE 1:** Baseline patients and implant characteristics.

	Total	Control	Test	*p*
N. Patients	59			
Gender				
Male	32 (54.2%)			
Female	27 (45.8%)			
Age	65.4 ± 8.7			
Smoking	7 (11.9%)			
History of periodontitis	38 (64.4)			
FMPS (%)	11.4 ± 5.2			
FMBS (%)	11.0 ± 5.5			
N. implants				
2	33 (55.9%)			
4	21 (35.6%)			
6	4 (6.8%)			
8	1 (1.7%)			
N. implants	182	91	91	
Position				
Anterior	23 (12.6%)	11 (12.1%)	12 (13.2%)	0.432
Premolar	81 (44.5%)	37 (40.7%)	44 (48.4%)	
Molar	78 (42.9%)	43 (47.3%)	35 (38.5%)	
ARCH				
Lower	92 (50.5%)	50 (54.9%)	42 (46.2%)	0.747
Upper	90 (49.5%)	41 (45.1%)	49 (53.8%)	
PI T0	56.4 ± 32.2	56.8 ± 32.3	56.0 ± 32.3	0.813
BoP T0	50.6 ± 28.8	48.9 ± 27.6	52.4 ± 29.9	0.264
PPD T0	3.25 ± 0.82	3.18 ± 0.83	3.32 ± 0.82	0.057
N. sites < 3 mm T0	3.92 ± 1.70	4.07 ± 1.69	3.78 ± 1.70	0.125
N. sites 4–5 mm T0	1.67 ± 1.39	1.55 ± 1.35	1.79 ± 1.42	0.136
N. sites > 6 mm T0	0.36 ± 0.65	0.32 ± 0.58	0.41 ± 0.71	0.163
PISTD T0	0.05 ± 0.33	0.07 ± 0.36	0.04 ± 0.29	0.317
KMW T0	0.71 ± 1.12	0.53 ± 1.12	0.90 ± 1.09	0.001**
CAL T0	3.23 ± 0.79	3.15 ± 0.77	3.32 ± 0.81	0.004**

*Note:* **p < 0.01. *N* (%) and mean ± SD. Results of Wald's chi2 test from simple binary logistic and linear regressions using GEE model (*p*‐values).

Abbreviations: BoP, bleeding on probing (%); CAL, clinical attachment level (mm); FMBS, Full mouth plaque score (%); FMPS, Full mouth plaque score (%); KMW, Keratinized mucosal width (mm); PI, plaque index (%); PISTD, peri‐implant soft‐tissue dehiscence (mm); PPD, probing depth (mm).

### 3.2 Clinical Outcomes

Between T0 to T1, BoP (primary outcome measure) decreased significantly only in test group from 52.4 (95% CI 46.2–58.6) to 33.7 (95% CI 27.2–40.2) (Δ: 18.7; *p* < 0.001), while statistically significant improvements between both T2 and T3 compared to T0 (*p* < 0.001) were detected in both groups. Nevertheless, differences between groups were not statistically significant at any time point (T1–T3) (*p* = 1.000). PPD reduction was statistically significant greater in the test (3.32; 95% CI 3.15–3.49) compared to the control group (T0–T1) (Δ: 0.18; *p* = 0.021). A similar trend was noticed also at T2 and T3. Difference between groups did not reach statistically significant difference at any time point (T2 *p* = 1.000; T3 = 0.123). A similar trend was noticed with respect to mean CAL reduction: at T3, PPD was 3.01 (95% CI 2.90–3.21) within the test and 2.87 (95% CI 2.75–3.01) in the control group (*p* = 0.123). From T0 to T3, PI dropped from 56.0 (95% CI 3.15–3.49) (Test) and 56.8 (95% CI 3.01–3.36) (Control) to 36.1 (95% CI 2.90–3.21) and 33.7 (95% CI 2.75–3.01), respectively (*p* = 1.000). Suppuration was never detected at any time point.

### 3.3 Treatment Success and Disease Resolution

Treatment success at T2 was observed in 58.2% (95% CI 47.9–68.6) of test and 56.0% (95% CI 45.7–66.4) control implants, with no significant difference between groups (*p* = 0.704). At T3, it was observed in 64.8% (95% CI 54.8–74.8) of test and 58.2% (95% CI 47.9–68.6) of control implants, with no statistically significant difference (*p* = 0.281) (Table 2). The frequency distribution of complete (BoP = 0) and partial (BoP ≤ 1, ≤ 2, ≤ 3) disease resolution at T2 did not significantly differ between groups (*p* > 0.05) (Table 3).

**TABLE 2:** Changes in clinical parameters at implant‐level at follow‐up visits for both groups.

	T0	T1	ΔT0–T1	T2	ΔT0–T2	T3	ΔT0–T3
BoP (%)							
Control	48.9 (43.1–54.7)	34.1 (27.7–40.4)	14.8; *p* = 0.053	28.0 (22.0–34.0)	20.9; *p* < 0.001***	24.2 (19.1–29.3)	24.7; *p* < 0.001***
Test	52.4 (46.2–58.6)	33.7 (27.2–40.2)	18.7; *p* < 0.001***	26.0 (20.5–31.5)	26.4; *p* < 0.001***	20.5 (16.0–25.1)	31.9; *p* < 0.001***
*p*	1	1		1		1	
PPD (mm)							
Control	3.18 (3.01–3.36)	3.06 (2.90–3.21)	0.13; *p* = 1	2.96 (2.83–3.10)	0.22; *p* = 0.561	2.88 (2.75–3.01)	0.30; *p* = 0.001**
Test	3.32 (3.15–3.49)	3.13 (2.97–3.29)	0.18; *p* = 0.021*	3.04 (2.90–3.17)	0.28; *p* = 0.023*	3.06 (2.90–3.21)	0.26; *p* = 0.028*
*p*	1	1		1		0.123	
CAL (mm)							
Control	3.15 (3.01–3.36)	3.06 (2.90–3.21)	0.09; *p* = 1	2.97 (2.83–3.10)	0.18; *p* = 0.561	2.87 (2.75–3.01)	0.27; *p* = 0.002**
Test	3.32 (3.15–3.49)	3.16 (2.97–3.29)	0.16; *p* = 0.075	3.04 (2.90–3.17)	0.28; *p* = 0.019*	3.01 (2.90–3.21)	0.31; *p* = 0.008**
*p*	1	1		1		0.123	
PI (%)							
Control	56.8 (3.01–3.36)	47.4 (2.90–3.21)	9.3; *p* = 0.378	36.6 (2.83–3.10)	20.1; *p* < 0.001***	33.7 (2.75–3.01)	23.0; *p* < 0.001***
Test	56.0 (3.15–3.49)	41.9 (2.97–3.29)	14.1; *p* = 0.021*	33.9 (2.90–3.17)	22.2; *p* < 0.001***	36.1 (2.90–3.21)	20.0; *p* < 0.001***
*p*	1	1		1		1	
KMW (mm)							
Control	0.53 (3.01–3.36)	0.58 (2.90–3.21)	−0.05; *p* = 1	0.66 (2.83–3.10)	−0.13; *p* = 1	0.73 (2.75–3.01)	−0.20; *p* = 1
Test	0.90 (3.15–3.49)	0.86 (2.97–3.29)	0.04; *p* = 1	0.82 (2.90–3.17)	0.08; *p* = 1	1.07 (2.90–3.21)	−0.16; *p* = 1
*p*	0.028*	0.172		1		0.942	

				T2		T3	ΔT3–T2
Success (%)							
Control				56.0 (45.7–66.4)		58.2 (47.9–68.6)	*p* = 0.705
Test				58.2 (47.9–68.6)		64.8 (54.8–74.8)	*p* = 0.303
*p*				0.704		0.281	

*Note:* *p < 0.05; **p < 0.01; ***p < 0.001. Mean (95% CI). Results of multiple pairwise comparisons (intra and inter‐groups) with Bonferroni's correction.

Abbreviations: BoP, bleeding on probing (%); CAL, clinical attachment level (mm); KMW, Keratinized mucosal width (mm); PI, plaque index (%); PPD, probing depth (mm).

**TABLE 3:** Frequency distribution of complete (BoP = 0) and partial (BoP ≤ = 1; ≤ 2; ≤ 3) disease resolution at T2 in both treatment groups.

	Control (*n* = 91)	Test (*n* = 91)	*p*
BoP = 0	32 (35.2%)	30 (33.0%)	0.741
BoP ≤ 1	52 (57.1%)	53 (58.2%)	0.850
BoP ≤ 2	62 (68.1%)	68 (74.7%)	0.238
BoP ≤ 3	75 (82.4%)	79 (86.8%)	0.361

*Note:* Results of Wald's chi2 test from simple binary logistic regression models using GEE.

Abbreviation: BoP, bleeding on probing (%).

### 3.4 Regression Models

The simple linear regression models for the clinical parameters detected a significant effect with respect to the variable “time” (BoP, PPD, PI, CAL), as well as for the variable “group” (PPD, CAL, KMW). However, the combination of the two failed to detect any significant effect (*p* > 0.05) (Table 4). The developed simple and multiple regression model revealed smoking (*p* = 0.008), implant position (i.e., premolar; *p* = 0.009) statistically significant affecting the primary outcome measure. While T0 PD ≥ 4 mm was only positively affecting the outcomes in the simple model (*p* = 0.043) (Table 5).

**TABLE 4:** Regression model with respect to the clinical parameters at implant‐level over the follow‐up visits for both groups.

	Time	Group	Time × Group
BoP (%)	< 0.001***	0.846	0.433
PPD (mm)	< 0.001***	0.034*	0.393
CAL (mm)	< 0.001***	0.031*	0.641
PI (%)	< 0.001***	0.357	0.269
KMW (mm)	0.644	0.001*	0.426
Treatment Success (%)	0.326	0.306	0.584

*Note:* *p < 0.05; ***p < 0.001. Results of Wald's chi2 test from the simple linear regression models using GEE for BoP, PPD, PI, KMW, and CAL. Simple binary logistic regression models using GEE for the variable Treatment Success.

Abbreviations: BoP, bleeding on probing (%); CAL, clinical attachment level (mm); KMW, Keratinized mucosal width (mm); PI, plaque index (%); PPD, probing depth (mm).

**TABLE 5:** Overall BoP change (T0–T3) by independent variables at patient and implant‐level.

	Simple			Multiple		
	Beta	95% CI	*p*	Beta	95% CI	*p*
Group						
Control	0			0		
Test	7.13	−0.94 to 15.2	0.083	6.22	−1.45 to 13.9	0.112
Gender						
Male	0					
Female	9.42	−4.19 to 23.0	0.175			
Age	0.45	−0.32 to 1.23	0.254			
Smoking						
No	0			0		
Yes	−24.0	−41.6 to −6.42	0.007**	−22.8	5.96–40.2	0.008**
History of perio						
No	0					
Yes	6.18	−6.93 to 19.3	0.356			
FMPS (%)	0.91	−0.33 to 2.16	0.151			
FMBS (%)	0.84	−0.45 to 2.12	0.202			
Position			< 0.001***			0.021*
Anterior	0			0		
Premolar	−14.3	−21.2 to −7.45	< 0.001***	−11.8	−20.5 to −2.99	0.009**
Molar	−7.96	−16.9 to 1.00	0.082	−4.73	−14.8 to 5.37	0.358
Arch						
Lower	0					
Upper	−3.01	−12.9 to 6.88	0.551			
PPD T0						
≤ 4 mm	0			0		
> 4 mm	13.8	0.41–27.2	0.043*	12.5	−0.01 to 25.0	0.050
KMW T0	3.33	−0.93 to 7.58	0.125	1.95	2.14 to −2.26	0.364

*Note:* *p < 0.05; **p < 0.01; ***p < 0.001. Results of simple and multiple linear regression using GEE model: beta coefficient and 95% CI.

Abbreviations: FMBS Full mouth plaque score (%); FMPS, Full mouth plaque score (%); KMW, Keratinized mucosal width (mm); PPD, probing depth (mm).

Details of the regression models for PPD, CAL, and PI are reported in Table S1–S3.

## 4 Discussion

The aim of this split‐mouth randomized clinical trial was to assess the potential benefits of chlorhexidine and xanthan‐based (XanCHX) gel as an adjunct to professional mechanical plaque removal (PMPR) in patients with PM. The results did not reveal any statistical differences in clinical outcomes after 3 months of follow‐up. Therefore, the null hypothesis could not be rejected. These findings are consistent with the systematic reviews by Dommisch et al. [34] and by Ye et al. [35], which similarly concluded that the adjunctive professional application of CHX leads no additional benefit in reducing BoP compared with PMPR alone. However, it should be underlined that all included studies evaluated several chlorhexidine formulations but not the one investigated in this trial (i.e., XanCHX). The overall improvement observed in terms of clinical outcomes within the whole observation period underscores the critical role of PMPR as the primary therapeutic intervention for PM [1, 10]. These results align with a randomized double‐blind clinical trial [36] demonstrating that non‐surgical mechanical debridement, with or without the adjunctive use of antiseptics, led to a reduction in BoP and PPD over the 3‐month follow‐up period, with the most significant improvements observed within the first month. When critically evaluate BoP change, primary outcome measure, it should be emphasized that the significant reduction in BoP in the test group at T1 aligns with the results provided by Gennai et al. [12], showing a beneficial effect of antiseptics administration on peri‐implant inflammatory parameters at within the first months. On the other hand, Ramanauskaite et al. [14] and Barootchi et al. [37] concluded that adjunctive measures did not lead to improvements in BoP compared to PMPR alone. These discrepancies may be attributed to potential biases, such as differences in PM case definition [14, 36], lack of standardization in outcome reporting [14, 36], and the absence of control groups [14]. It is also important to emphasize that none of these studies reported the use of XanCHX gel as an adjunctive measure.

The rationale of adopting XanCHX for PM treatment was translated from the treatment of chronic periodontitis, showing that the use of gel along with PMPR significantly improved BoP outcomes between baseline and 3 months [38]. These results were confirmed within a recent systematic review with meta‐analysis reporting clinical improvement in terms of PPD and CAL gain [39]. More specifically, when compared to the application of CHX gel, the formulation used within this trial revealed greater PPD reduction [19].

One important endpoint in the assessment of PM treatment efficacy is the evaluation of “treatment success:” in the present investigation, applying as threshold the presence of maximum one site BoP+ [1], 58% vs. 56% of implants were considered “successfully treated.” These results are consistent with previous reports which have consistently remarked the limited resolution rate of PM following non‐surgical treatment and quantified in roughly 50% the possibility of achieving “treatment success” [12, 14, 37]: more specifically, Clementini et al. reported at 6‐month after therapy a treatment success of 66.13% (test group) and 65.52% (control group) [33], as well as Nicola et al. who highlighted 3‐month treatment success of 62.13% vs. 54.54% in the test and control group, respectively [40]. When it comes to systematic reviews, Verket et al. [41], observed that treatment success rates after mechanical/physical instrumentation decreased markedly over time, dropping from 30.9%–34.5% at 3 months to only 8.3%–16.7% at 6 months. Such findings underscore that long‐term stability cannot rely solely on the initial therapeutic approach; rather, it critically depends on patient compliance, both in maintaining adequate home oral hygiene and in adhering to regular supportive peri‐implant care (SPIC) protocols. The significant increase in shallow probing pockets (sites < 3 mm) observed between T0 and T4 in the overall sample is consistent with previous findings. As anticipated, no significant changes were noticed with respect to PISTD and KMW. These are, in fact, important peri‐implant soft‐tissue parameters whose change which might be important on the longitudinal assessment of disease severity and on the consequent onset of peri‐implantitis [41]. More specifically, the lack peri‐implant keratinized tissue has been linked to a higher incidence of peri‐implantitis, increased plaque accumulation, soft tissue recession, increased patients' discomfort during brushing procedures and increased risk for peri‐implantitis [14, 42, 43]. It is in light of the increased evidence that some authors have proposed peri‐implant soft‐tissue phenotype modification by means of a free gingival graft with positive clinical outcomes in terms of PM treatment [44]. When focusing on the factors potentially related to treatment success, smoking appears to have a direct influence on it. This observation is consistent with previous data [45], supporting the hypothesis that smokers present poorer peri‐implant conditions.

The present study does present elements of strengths, such as being, to the authors' knowledge, the first controlled clinical trial on the use of XanCHX gel as an adjunct to PMPR in the treatment of PM as well as the split mouth nature. Furthermore, the sample size is adequate to test the research hypothesis and is in line with previous similar studies.

However, some limitations should be disclosed: the absence of blinding as well the lack of the application of a control gel, may have introduced potential bias. Another aspect to be considered was the lack of prosthesis removal: indeed, effective access for biofilm removal around implant‐supported prostheses is crucial for both the prevention and management of peri‐implant diseases [2]. Implants with restoration margins positioned above the mucosal line showed significantly greater reductions in probing depths after PM treatment, compared to those with margins placed below the mucosa [36]. However, on this aspect, it should be underlined that all reconstruction fitting and cleanability was judged clinically satisfactory. Therefore, no corrections were needed nor performed. Furthermore, an additional qualitative evaluation of bleeding on probing (mBI [46]) would have provided additional information, resulting in a more comprehensive clinical assessment. Nevertheless, it must be recalled that the decision of using a dichotomous evaluation was based on be rationale of being adherent to the most commonly system within a “real‐life” clinical scenario. Furthermore, patient‐reported outcome measures (PROMS) were not evaluated. In conclusion, although the addition of Xan‐CHX gel to PMPR alone in the treatment of PM did result in better clinical outcomes, its application contributed to a greater short‐term BoP reduction. Further investigations are needed to investigate its efficacy in more severe cases (i.e., PM with suppuration).

## 5 Conclusions

Within the limitations of the present study, the adjunctive use of XanCHX gel did not result in any statistically significant clinical benefit compared to PMPR alone in the treatment of PM up to 6 months, despite the reported clinical positive effects within the first month after treatment.

## Author Contributions

J.C. and J.L. contributed to study conception, performed the treatments and contributed to the writing; A.C. and U.G. contributed to study conception, data collection and critically revised the manuscript; G.A. performed the statistical analysis; A.R. interpreted the data and led to the writing; F.P. critically revised the manuscript. All authors agreed on the final draft of the manuscript.

## Funding

The authors have nothing to report.

## Conflicts of Interest

The authors declare no conflicts of interest.

## Supporting information


**Table S1:** Overall PD change (T0–T3) by independent variables at patient and implant‐level.
**Table S2:** Overall PI change (T0–T3) by independent variables at patient and implant‐level.
**Table S3:** Overall CAL change (T0–T3) by independent variables at patient and implant‐level.

## Data Availability

The data that support the findings of this study are available on request from the corresponding author. The data are not publicly available due to privacy or ethical restrictions.
