# Supplementary material for: Efficacy of Xanthan‐Based Chlorhexidine Gel in Peri‐Implant Mucositis Treatment: A Split‐Mouth Randomized Clinical Trial
Source: Clin Implant Dent Relat Res. 2026 Mar 21;28(2):e70137. doi: 10.1111/cid.70137 (PMC13005090; doi:10.1111/cid.70137)
Supplement: Supplementary file 1 — Table S1: Overall PD change (T0–T3) by independent variables at patient and implant‐level. Table S2: Overall PI change (T0–T3) by independent variables at patient and implant‐level. Table S3: Overall CAL change (T0–T3) by independent variables at patient and implant‐level. [file CID-28-0-s001.docx]

**Supplementary materials**

**Table S1.** Overall PD change (T0-T3) by independent variables at patient and implant-level

|  | **SIMPLE** | | |  | **MULTIPLE** | | |
| --- | --- | --- | --- | --- | --- | --- | --- |
|  | **Beta** | **95% CI** | **p-value** |  | **Beta** | **95% CI** | **p-value** |
| **GROUP** |  |  |  |  |  |  |  |
| Control | 0 |  |  |  | 0 |  |  |
| Test | -0.04 | -0.23 0.14 | 0.657 |  | -0.09 | -0.25 0.07 | 0.248 |
| **GENDER** |  |  |  |  |  |  |  |
| Male | 0 |  |  |  |  |  |  |
| Female | 0.01 | -0.22 0.24 | 0.906 |  |  |  |  |
| **AGE** | -0.01 | -0.02 0.01 | 0.542 |  |  |  |  |
| **SMOKING** |  |  |  |  |  |  |  |
| No | 0 |  |  |  |  |  |  |
| Yes | -0.02 | -0.24 0.19 | 0.829 |  |  |  |  |
| **HISTORY OF PERIO** |  |  |  |  |  |  |  |
| No | 0 |  |  |  |  |  |  |
| Yes | -0.15 | -0.39 0.10 | 0.251 |  |  |  |  |
| **FMPS (%)** | 0.02 | -0.01 0.04 | 0.116 |  |  |  |  |
| **FMBS (%)** | 0.01 | -0.01 0.03 | 0.469 |  |  |  |  |
| **POSITION** |  |  | 0.539 |  |  |  |  |
| Anterior | 0 |  |  |  |  |  |  |
| Premolar | -0.13 | -0.41 0.15 | 0.357 |  |  |  |  |
| Molar | -0.01 | -0.28 0.25 | 0.930 |  |  |  |  |
| **ARCH** |  |  |  |  |  |  |  |
| Lower | 0 |  |  |  |  |  |  |
| **Upper** | 0.02 | -0.19 0.24 | 0.839 |  |  |  |  |
| **PPD T0** |  |  |  |  |  |  |  |
| <=4mm | 0 |  |  |  | 0 |  |  |
| >4mm | 0.85 | 0.57 1.13 | **<0.001**** |  | 0.86 | 0.58 1.14 | **<0.001***** |
| **KMW T0** | 0.02 | -0.06 0.11 | 0.586 |  | 0.03 | -0.05 0.11 | 0.431 |

**p<0.01; ***p<0.001

Note: Results of simple and multiple linear regression using GEE model: beta coefficient and 95%CI.

Abbreviations: FMPS, Full mouth plaque score (%); FMBS Full mouth plaque score (%); PPD, probing depth (mm); KMW, Keratinized mucosal width (mm).

**Table S2.** Overall PI change (T0-T3) by independent variables at patient and implant-level

|  | **SIMPLE** | | |  | **MULTIPLE** | | |
| --- | --- | --- | --- | --- | --- | --- | --- |
|  | **Beta** | **95% CI** | **p-value** |  | **Beta** | **95% CI** | **p-value** |
| **GROUP** |  |  |  |  |  |  |  |
| Control | 0 |  |  |  | 0 |  |  |
| Test | -3.11 | -10.8 4.61 | 0.430 |  | -2.54 | -10.2 5.16 | 0.518 |
| **GENDER** |  |  |  |  |  |  |  |
| Male | 0 |  |  |  | 0 |  |  |
| Female | 19.9 | 4.33 35.6 | **0.012*** |  | 18.9 | 3.79 34.0 | **0.014*** |
| **AGE** | 0.71 | -0.32 1.74 | 0.177 |  |  |  |  |
| **SMOKING** |  |  |  |  |  |  |  |
| No | 0 |  |  |  |  |  |  |
| Yes | -17.9 | -47.7 11.7 | 0.235 |  |  |  |  |
| **HISTORY OF PERIO** |  |  |  |  |  |  |  |
| No | 0 |  |  |  | **0** |  |  |
| Yes | -13.7 | -29.6 2.18 | 0.091 |  | -13.3 | -28.2 1.50 | 0.078 |
| **FMPS (%)** | -0.44 | -1.91 1.04 | 0.559 |  |  |  |  |
| **FMBS (%)** | -0.55 | -2.04 0.94 | 0.467 |  |  |  |  |
| **POSITION** |  |  | 0.158 |  |  |  |  |
| Anterior | 0 |  |  |  |  |  |  |
| Premolar | 0.87 | -16.9 18.7 | 0.924 |  |  |  |  |
| Molar | 11.3 | -7.54 30.1 | 0.240 |  |  |  |  |
| **ARCH** |  |  |  |  |  |  |  |
| Lower | 0 |  |  |  | 0 |  |  |
| Upper | -14.0 | -27.4 -0.68 | **0.039*** |  | -13.2 | -25.7 -0.73 | **0.038*** |
| **PD T0** |  |  |  |  |  |  |  |
| <=4mm | 0 |  |  |  | 0 |  |  |
| >4mm | -1.38 | -11.9 9.20 | 0.798 |  | 1.13 | -8.93 11.2 | 0.826 |
| **KMW T0** | -0.04 | -5.09 5.01 | 0.988 |  | 1.11 | -4.08 6.30 | 0.675 |

*p<0.05

Note: Results of simple and multiple linear regression using GEE model: beta coefficient and 95%CI.

Abbreviations: FMPS, Full mouth plaque score (%); FMBS Full mouth plaque score (%); PPD, probing depth (mm); KMW, Keratinized mucosal width (mm).

**Table S3.** Overall CAL change (T0-T3) by independent variables at patient and implant-level

|  | **SIMPLE** | | |  | **MULTIPLE** | | |
| --- | --- | --- | --- | --- | --- | --- | --- |
|  | **Beta** | **95% CI** | **p-value** |  | **Beta** | **95% CI** | **p-value** |
| **GROUP** |  |  |  |  |  |  |  |
| Control | 0 |  |  |  | 0 |  |  |
| Test | 0.04 | -0.12 0.20 | 0.622 |  | 0.01 | -0.12 0.14 | 0.908 |
| **GENDER** |  |  |  |  |  |  |  |
| Male | 0 |  |  |  |  |  |  |
| Female | 0.07 | -0.20 0.34 | 0.624 |  |  |  |  |
| **AGE** | -0.01 | -0.02 0.01 | 0.188 |  |  |  |  |
| **SMOKING** |  |  |  |  |  |  |  |
| No | 0 |  |  |  |  |  |  |
| Yes | -0.06 | -0.30 0.18 | 0.621 |  |  |  |  |
| **HISTORY OF PERIO** |  |  |  |  |  |  |  |
| No | 0 |  |  |  |  |  |  |
| Yes | -0.26 | -0.61 0.09 | 0.141 |  |  |  |  |
| **FMPS (%)** | 0.02 | -0.01 0.04 | 0.129 |  |  |  |  |
| **FMBS (%)** | 0.01 | -0.01 0.03 | 0.371 |  |  |  |  |
| **POSITION** |  |  | 0.385 |  |  |  |  |
| Anterior | 0 |  |  |  |  |  |  |
| Premolar | -0.15 | -0.44 0.14 | 0.304 |  |  |  |  |
| Molar | 0.00 | -0.29 0.29 | 0.977 |  |  |  |  |
| **ARCH** |  |  |  |  |  |  |  |
| Lower | 0 |  |  |  |  |  |  |
| **Upper** | -0.02 | -0.26 0.22 | 0.886 |  |  |  |  |
| **PD T0** |  |  |  |  |  |  |  |
| <=4mm | 0 |  |  |  |  |  |  |
| >4mm | 0.68 | 0.45 0.91 | **<0.001***** |  | 0.68 | 0.45 0.91 | **<0.001***** |
| **KMW T0** | 0.00 | -0.07 0.07 | 0.945 |  | -0.01 | -0.07 0.07 | 0.959 |

***p<0.001

Note: Results of simple and multiple linear regression using GEE model: beta coefficient and 95%CI.

Abbreviations: FMPS, Full mouth plaque score (%); FMBS Full mouth plaque score (%); PPD, probing depth (mm); KMW, Keratinized mucosal width (mm).
